# Erratum: Connecting genetic risk to disease end points through the human blood plasma proteome

**DOI:** 10.1038/ncomms15345

**Published:** 2017-04-11

**Authors:** Karsten Suhre, Matthias Arnold, Aditya Mukund Bhagwat, Richard J. Cotton, Rudolf Engelke, Johannes Raffler, Hina Sarwath, Gaurav Thareja, Annika Wahl, Robert Kirk DeLisle, Larry Gold, Marija Pezer, Gordan Lauc, Mohammed A. El-Din Selim, Dennis O. Mook-Kanamori, Eman K. Al-Dous, Yasmin A. Mohamoud, Joel Malek, Konstantin Strauch, Harald Grallert, Annette Peters, Gabi Kastenmüller, Christian Gieger, Johannes Graumann

Nature Communications 8: Article number: 14357; DOI: 10.1038/ncomms14357 (2017); Published 02 27 2017; Updated 04 11 2017

The original version of the Supplementary Information attached to this Article did not include Supplementary Note 1 The HTML has now been updated to include a corrected version of the Supplementary Information.

## 

