# Supplementary material for: Connecting genetic risk to disease end points through the human blood plasma proteome
Source: Nat Commun. 2017 Feb 27;8:14357. doi: 10.1038/ncomms14357 (PMC5333359; doi:10.1038/ncomms14357)
Supplement: Supplementary Information — Supplementary Figures, Supplementary Tables, Supplementary Note, and Supplementary References [file ncomms14357-s1.pdf]

Supplementary Table 1: Published *cis*-pQTL studies in human blood.

Study population, number of samples, protein features tested, *cis*-pQTLs found, and technique used for previously published *cis*-pQTL studies.

Study	Study population	Samples	Proteins	*Cis*-pQTLs	Technique
Melzer *et al*. (2008) [1]	Population study	1,200	42	8	Immuno-assay
Lourdusamy *et al*. (2012) [2]	Elderly Europeans	96	778	60[+]	Aptamer-based
Johansson *et al*. (2013) [3]	Two population cohorts	1,060	163	5	MS
Kim *et al*. (2013) [4]	Alzheimer cohort	521	132	28	Immuno-assay
Enroth *et al*. (2014) [5]	Population study	970	77	18	Immuno-assay
Liu *et al*. (2015) [6]	Female twins	113	342	18[#]	MS

[+]reported at 5% FDR, [#]including one *trans*-pQTL

Supplementary Table 2: Replication of pQTLs from GWAS studies with ELISA assays.

Several GWAS with bio-medically relevant protein assays have been published. Here we report associations that match *cis*-eQTLs in our study (Locus number). In cases where the GWAS variant did not match the sentinel SNP of our association, we report the GWAS SNP identifier and the linkage disequilibrium r^2.

Protein name	Protein	Locus	Tag SNP	Chr	Position	P-value	GWAS SNP (r^2)	Reference
Granulins	GRN	71	rs646776	1	109,818,530	1.0×10^{-52}	same SNP	Carrasquillo *et al.* [7]
Interleukin-6 receptor subunit alpha	IL6R	2	rs4129267	1	154,426,264	1.6×10^{-265}	rs2228145 (1.0)	Van Dongen *et al.* [8]
P-Selectin	SELP	158	rs6136	1	169,563,951	1.1×10^{-25}	same SNP	Barbalic *et al.* [9]
Interleukin-1 receptor-like 1	IL1RL1	44	rs12712135	2	102,930,948	4.4×10^{-76}	rs950880 (0.53)	Ho *et al.* [10]
Adiponectin	ADIPOQ	-	rs17366568	3	186,570,453	6.2×10^{-7}	same SNP	Heid *et al.* [11]
Insulin-like growth factor-binding protein 3	IGFBP3	178	rs2949833	7	45,984,820	2.6×10^{-22}	rs11977526 (0.59)	Kaplan *et al.* [12]
von Willebrand factor	VWF	36	rs612169	9	136,143,442	1.2×10^{-20}	rs657152 (0.89)	Zabaneh et al. [13]
sE-Selectin	SELE	28	rs651007	9	136,153,875	1.3×10^{-96}	same SNP	Qi et al. [14,15]
sP-Selectin	SELP	28	rs651007	9	136,153,875	7.4×10^{-13}	rs579459 (1.0)	Barbalic *et al.* [9]
Intercellular adhesion molecule 1	ICAM1	-	rs651007[+]	9	136,153,875	3.5×10^{-7}	same SNP	Paré et al. [16]
Serum amyloid A-1 protein	SAA1	22	rs1993373	11	18,280,635	2.2×10^{-105}	rs4638289 (0.99)	Marzi *et al.* [17]
Coagulation Factor VII	F7	58	rs776905	13	113,781,942	9.2×10^{-65}	rs561241 (0.53)	Yang *et al.* [18]
Haptoglobin	HP	46	rs2000999	16	72,114,002	7.4×10^{-45}	same SNP	Froguel *et al.* [19]
Intercellular adhesion molecule 1	ICAM1	1	rs5498	19	10,395,683	7.7×10^{-267}	same SNP	Paré et al. [16]
Cystatin-C	CST3	293	rs6036478	20	23,611,359	9.6×10^{-15}	rs1158167 (0.94)	Hwang *et al.* [20]
Vitamin K-dependent protein C	PROC	106	rs867186	20	33,764,554	5.5×10^{-38}	same SNP	Tang *et al.* [21]

[+]Association p-value computed including *cis*-pQTL SNP rs5498 as additional co-variate to account for strong *cis*-encoded variance in ICAM1.

Supplementary Table 3: Replication of *cis*-pQTLs in an independent study with the SomaLogic assay.

Using the SomaLogic platform to analyse blood plasma samples of 100 individuals, Lourdusamy et al. [2] reported 60 *cis*-pQTLs at a false discovery rate of 5%. Here we report P-values from the KORA study using matching imputed SNPs, or, when these were not available, using a suitable tagging SNP (r^2>0.5, indicated below the P-value). Twelve associations lacked a suitable tagging SNP. A SNP-protein association was considered replicated if p<0.05/120, conservatively correcting for 60 tests on imputed and tagging SNPs. 34 of 48 associations replicated, including the twenty strongest associations on the list.

Protein	SNP	Chr	Position	P-value (Lourdusamy)	P-value (this study)	Replicated
Tenascin (TNC)	rs7021589	9	117,804,667	1.50E-17	1.77E-63	YES
Sialic acid-binding Ig-like lectin 9 (SIGLEC9)	rs2075803	19	51,628,529	9.63E-17	7.13E-122	YES
Platelet-derived growth factor receptor beta (PDGFRB)	rs2240781	5	149,516,480	4.42E-15	4.71E-161	YES
Low affinity immunoglobulin gamma Fc region receptor II-a/b (FCGR2A FCGR2B)	rs1801274	1	161,479,745	5.17E-15	1.24E-120	YES
Myeloid cell surface antigen CD33 (CD33)	rs12985029	19	51,713,365	2.65E-13	1.76E-66	YES
Teratocarcinoma-derived growth factor 1 (TDGF1)	chr3:46619238	3	46,619,238	7.10E-13	3.78E-145 (rs3806701)	YES
Interleukin-1 Receptor accessory protein (IL1RAP)	rs724608	3	190,348,810	8.81E-13	1.16E-143	YES
Interleukin-6 receptor subunit alpha (IL6R)	rs11265613	1	154,418,415	1.43E-12	1.25E-239	YES
Low affinity immunoglobulin gamma Fc region receptor II-a/b (FCGR2A FCGR2B)	rs6665610	1	161,641,384	1.27E-11	4.26E-160	YES
C-C motif chemokine 16 (CCL16)	chr17:34306470	17	34,306,470	2.52E-11	1.91E-95 (rs33995560)	YES
Serum amyloid A-1 protein (SAA1)	chr11:18290906	11	18,290,906	1.16E-10	2.17E-105 (rs1993373)	YES
CD109 antigen (CD109)	rs9447004	6	74,458,737	8.61E-10	1.56E-44	YES
Mannose-binding protein C (MBL2)	rs7899547	10	54,536,839	1.60E-9	2.44E-102	YES
Low molecular weight phosphotyrosine protein phosphatase (ACP1)	rs17713879	2	254,215	2.46E-9	2.04E-215	YES
Coagulation Factor VII (F7)	rs474671	13	113,776,218	2.75E-9	5.62E-65	YES

Complement C2 (C2)	chr6:31981247	6	31,981,247	3.70E-9	n/a	
Complement factor H (CFH)	rs1048663	1	196,674,982	2.94E-8	4.53E-25	YES
Chitotriosidase-1 (CHIT1)	rs2486950	1	203,174,670	3.15E-8	7.82E-111	YES
Ephrin type-A receptor 1 (EPHA1)	rs1804527	7	143,088,823	4.34E-8	4.50E-67	YES
A disintegrin and metalloproteinase with thrombospondin motifs 13 (ADAMTS13)	rs28647808	9	136,305,530	5.45E-8	2.61E-42	YES
Alpha-2-HS-glycoprotein (AHSG)	rs2070632	3	186,334,004	7.31E-8	2.00E-34	YES
Interleukin-22 (IL22)	rs7133527	12	68,357,125	4.60E-7	8.02E-1	NO
Interleukin-17 receptor A (IL17RA)	rs2241047	22	17,586,583	6.35E-7	3.99E-96	YES
26S proteasome non-ATPase regulatory subunit 7 (PSMD7)	rs7359422	16	74,592,483	8.49E-7	1.46E-2	NO
Interleukin-1 receptor-like 1 (IL1RL1)	rs12470864	2	102,926,362	8.85E-7	2.96E-69	YES
Myeloblastin (PRTN3)	rs7251804	19	839,948	9.49E-7	8.30E-2	NO
Interleukin-12 receptor subunit beta-2 (IL12RB2)	chr1:67489003	1	67,489,003	9.82E-7	n/a	
Junctional adhesion molecule C (JAM3)	rs11223763	11	134,214,549	1.60E-6	8.43E-1	NO
Tumor-associated calcium signal transducer 2 (TACSTD2)	rs232815	1	59,080,879	2.18E-6	3.68E-1	NO
Endothelial monocyte-activating polypeptide 2 (AIMP1)	chr4:107298048	4	107,298,048	3.56E-6	0.022 (rs17275627)	NO
Complement factor H-related protein 5 (CFHR5)	rs6695321	1	196,675,861	4.13E-6	9.70E-20	YES
Granulysin (GNLY)	rs7577293	2	85,935,282	4.85E-6	4.70E-76	YES
Ectonucleoside triphosphate diphosphohydrolase 1 (ENTPD1)	rs72822592	10	97,180,509	5.54E-6	5.25E-1	NO
C-X-C motif chemokine 5 (CXCL5)	chr4:74596624	4	74,596,624	7.29E-6	n/a	
Interleukin-18 receptor 1 (IL18R1)	chr2:102951851	2	102,951,851	8.26E-6	1.09E-26 (rs1882510)	YES
Kininogen-1 (KNG1)	rs5030049	3	186,450,863	1.11E-5	1.37E-96	YES
Lymphotactin (XCL1)	rs1933112	1	168,521,417	1.33E-5	6.47E-7	YES
Cadherin-6 (CDH6)	rs13165280	5	31,578,552	1.39E-5	4.51E-1	NO
Phospholipase A2, membrane associated (PLA2G2A)	rs11573156	1	20,306,146	1.95E-5	9.64E-39	YES
C-C motif chemokine 15 (CCL15)	rs854624	17	34,327,923	2.00E-5	2.49E-119	YES

Microtubule-associated protein tau (MAPT)	rs73317026	17	44,081,268	2.12E-5	n/a	
Lymphotoxin alpha1:beta2 (LTA LTB)	rs9267054	6	31,397,367	2.21E-5	n/a	
Protein kinase C beta type (splice variant beta-II) (PRKCB)	rs7499480	16	23,825,207	2.95E-5	8.08E-1	NO
Interleukin-25 (IL25)	chr14:23770877	14	23,770,877	3.00E-5	n/a	
Apolipoprotein E (APOE)	rs2967668	19	45,302,951	4.15E-5	1.38E-4	YES
Kallikrein-5 (KLK5)	rs266863	19	51,355,650	4.50E-5	8.01E-2	NO
Tyrosine-protein kinase transmembrane receptor ROR1 (ROR1)	rs71499308	1	64,666,320	4.68E-5	n/a	
Lymphocyte antigen 86 (LY86)	rs9405302	6	6,477,849	5.10E-5	6.59E-1	NO
Interleukin-10 (IL10)	chr1:207234422	1	207,234,422	6.04E-5	n/a	
Parathyroid hormone-related protein (PTHLH)	chr12:27848777	12	27,848,777	6.25E-5	n/a	
Complement C4b (C4A C4B)	rs2844452	6	31,882,024	6.72E-5	n/a	
Cathepsin S (CTSS)	chr1:150868102	1	150,868,102	7.65E-5	9.49E-17 (rs267738)	YES
Intercellular adhesion molecule 1 (ICAM1)	chr19:10082034	19	10,082,034	9.87E-5	0.82 (rs12461154)	NO
Pyridoxal phosphate phosphatase (PDXP)	rs5995497	22	38,182,706	1.00E-4	2.17E-2	NO
Metalloproteinase inhibitor 3 (TIMP3)	rs2413151	22	33,167,869	1.07E-4	6.39E-100	YES
NADPH--cytochrome P450 reductase (POR)	rs11770797	7	75,855,511	1.09E-4	1.58E-6	YES
Carboxypeptidase B2 (CPB2)	rs2094247	13	46,602,162	1.17E-4	5.89E-73	YES
Matrix metalloproteinase-17 (MMP17)	rs10751701	12	132,330,735	1.21E-4	4.12E-1	NO

Supplementary Table 4: Replication of *cis*-pQTLs using the Myriad Rules Based Medicine Human DiscoveryMAP.

Using the multiplex immunoassay of the Myriad Rules Based Medicine (RBM) Human DiscoveryMAP panel v1.0 on the Lumine×100 platform to analyse samples from 521 participants of the ADNI cohort, Kim et al. [4] reported 28 *cis*-pQTLs at a significance level of $p<2.44\times10^{-5}$. (Table 3 in [4]). Here we report P-values from the KORA study for 17 matching imputed SNP-protein pairs. For two proteins no matching SNP was found, nine proteins were not covered by the SomaLogic panel. A SNP-protein association was considered replicated if p<0.05/17. Thirteen out of 17 associations replicated, including the eight strongest associations in the list.

Protein	SNP	Chr	Position	P-value (Kim et al.)	P-value (this study)	Replicated
Complement factor H (CFH)	rs6677604	1	196,686,918	9.29E-112	1.59E-13	YES
Complement factor H (CFH)	rs7517126	1	196,840,272	1.46E-60	7.61E-08	YES
Interleukin-6 receptor subunit alpha (IL6R)	rs4129267	1	154,426,264	1.56E-58	8.79E-266	YES
Alpha-2-HS-glycoprotein (AHSG)	rs2070633	3	186,335,941	4.50E-31	1.86E-38	YES
Apolipoprotein E (isoform E2) (APOE)	rs429358	19	45,411,941	7.27E-30	1.77E-12	YES
C-C motif chemokine 18 (CCL18)	rs854462	17	34,386,090	9.15E-24	2.96E-47	YES
Interleukin-16 (IL16)	rs4778636	15	81,591,639	5.08E-19	3.93E-27	YES
C-C motif chemokine 16 (CCL16)	rs11080369	17	34,305,164	8.03E-19	1.36E-92	YES
Angiotensinogen (AGT)	rs4762	1	230,845,977	4.74E-18	0.0301	NO
C-C motif chemokine 23 (CCL23)	rs854656	17	34,345,661	2.61E-13	2.26E-03	YES
C-C motif chemokine 8 (CCL8)	rs12602195	17	32,660,149	2.40E-12	3.79E-11	YES
Neuronal cell adhesion molecule (NRCAM)	rs10487849	7	107,987,085	1.36E-08	0.661	NO
Coagulation Factor VII (F7)	rs488703	13	113,770,876	2.00E-08	1.30E-63	YES
Alpha-1-antitrypsin (SERPINA1)	rs7151526	14	94,863,636	7.44E-07	0.0215	NO
Epidermal growth factor receptor (EGFR)	rs13244925	7	55,192,256	9.12E-07	0.0553	NO
Intercellular adhesion molecule 1 (ICAM1)	rs1799969	19	10,394,792	1.03E-06	7.90E-36	YES
Cystatin-C (CST3)	rs2424590	20	23,636,980	4.75E-06	3.78E-13	YES

Supplementary Table 5: Replication of *cis*-pQTLs using the Olink Proseek Multiplex Oncology I96x96 kit.

Using 92 proteins measured in blood plasma of 1,005 individuals with the Olink Proseek Multiplex Oncology I^{96x96} kit and quantified by real-time PCR using the Fluidigm BioMark HD real-time PCR, Enroth et al. [6] identified 23 *cis*-pQTLs (Table 3 in [6]). Here we report P-values from the KORA study for 18 matching imputed SNP-protein pairs. For one protein no matching SNP was found, four were not covered by the SomaLogic panel. A SNP-protein association was considered replicated if p<0.05/18. Twelve out of 18 associations were replicated.

Protein	SNP	Chr	Position	P-value (Enroth et al.)	P-value (this study)	Replicated
Interleukin-6 receptor subunit alpha (IL6R)	rs4129267	1	154,426,264	4.39E-58	8.79E-266	YES
C-X-C motif chemokine 10 (CXCL10)	rs11548618	4	76,943,947	6.78E-37	4.86E-21	YES
C-C motif chemokine 24 (CCL24)	rs6946822	7	75,479,448	2.02E-36	1.83E-01	NO
C-X-C motif chemokine 5 (CXCL5)	rs425535	4	74,863,997	4.27E-26	1.21E-09	YES
C-X-C motif chemokine 5 (CXCL5)	rs2472649	4	74,857,708	3.57E-21	1.80E-06	YES
interleukin-17 receptor B (IL17RB)	rs6801605	3	53,876,218	1.75E-18	7.48E-19	YES
Kallikrein-11 (KLK11)	rs117268623	19	51,527,970	5.25E-18	4.97E-03	NO
Melanoma-derived growth regulatory protein (MIA)	rs2230694	19	41,263,403	6.83E-17	9.69E-69	YES
Interleukin-12 (IL12A IL12B)	rs10045431	5	158,814,533	8.99E-17	9.03E-01	NO
Melanoma-derived growth regulatory protein (MIA)	rs2233159	19	41,283,365	1.07E-16	1.12E-69	YES
Melanoma-derived growth regulatory protein (MIA)	rs2607426	19	41,274,713	1.13E-16	1.05E-69	YES
Melanoma-derived growth regulatory protein (MIA)	rs2233154	19	41,281,346	1.13E-16	1.83E-69	YES
E-Selectin (SELE)	rs507666	9	136,149,399	5.01E-16	8.47E-118	YES
MHC class I polypeptide-related sequence A (MICA)	rs3869132	6	31,410,948	5.33E-16	1.12E-91	YES
C-C motif chemokine 19 (CCL19)	rs7775228	6	32,658,079	2.63E-13	3.21E-06	YES
C-C motif chemokine 24 (CCL24)	rs11465293	7	75,442,723	7.95E-13	4.71E-01	NO
MHC class I polypeptide-related sequence A (MICA)	rs2263316	6	31,421,297	1.02E-08	3.97E-03	NO
C-X-C motif chemokine 5 (CXCL5)	rs2393967	10	65,133,156	4.54E-08	2.87E-01	NO

Supplementary Table 6: Proteins with a replicated pQTL that have a biomarker application. Annotation as biomarker application derived from Ingenuity Pathway Analysis (IPA) database.

Target	Protein (Gene Symbol)	Biomarker Application(s)
6Ckine	C-C motif chemokine 21 (CCL21)	disease progression
Alkaline phosphatase, bone	Alkaline phosphatase, tissue-nonspecific isozyme (ALPL)	efficacy, safety
Angiogenin	Angiogenin (ANG)	response to therapy
annexin II	Annexin A2 (ANXA2)	diagnosis
BCAM	Basal Cell Adhesion Molecule (BCAM)	diagnosis
b-Endorphin	Beta-endorphin (POMC)	diagnosis, efficacy
bFGF	Fibroblast growth factor 2 (FGF2)	diagnosis, efficacy, prognosis
Cadherin-5	Cadherin-5 (CDH5)	diagnosis
CD23	Low affinity immunoglobulin epsilon Fc receptor (FCER2)	diagnosis
Chitotriosidase-1	Chitotriosidase-1 (CHIT1)	efficacy
Coagulation Factor V	Coagulation Factor V (F5)	diagnosis
Cripto	Teratocarcinoma-derived growth factor 1 (TDGF1)	diagnosis
ECM1	Extracellular matrix protein 1 (ECM1)	diagnosis
Fibronectin	Fibronectin (FN1)	diagnosis, efficacy, prognosis
G-CSF	Granulocyte colony-stimulating factor (CSF3)	diagnosis
Gro-a	Growth-regulated alpha protein (CXCL1)	disease progression
Haptoglobin, Mixed Type	Haptoglobin (HP)	diagnosis, efficacy
IGFBP-3	Insulin-like growth factor-binding protein 3 (IGFBP3)	diagnosis, disease progression, efficacy, prognosis, safety
IL-6 sRa	Interleukin-6 receptor subunit alpha (IL6R)	efficacy
IR	Insulin receptor (INSR)	prognosis
MBL	Mannose-binding protein C (MBL2)	diagnosis
Met	Hepatocyte growth factor receptor (MET)	diagnosis, disease progression, efficacy, prognosis, response to therapy
MIA	Melanoma-derived growth regulatory protein (MIA)	disease progression, prognosis
MICA	MHC class I polypeptide-related sequence A (MICA)	safety
MICB	MHC class I polypeptide-related sequence B (MICB)	safety
MIP-1a	C-C motif chemokine 3 (CCL3)	diagnosis, efficacy, prognosis

MMP-1	Interstitial collagenase (MMP1)	diagnosis, efficacy, safety
MMP-12	Macrophage metalloelastase (MMP12)	efficacy
MMP-8	Neutrophil collagenase (MMP8)	efficacy
Notch 1	Neurogenic locus notch homolog protein 1 (NOTCH1)	diagnosis, efficacy
NPS-PLA2	Phospholipase A2, membrane associated (PLA2G2A)	efficacy, prognosis, safety
PARC	C-C motif chemokine 18 (CCL18)	efficacy, safety
PDGF Rb	Platelet-derived growth factor receptor beta (PDGFRB)	prognosis, response to therapy
Protein C	Vitamin K-dependent protein C (PROC)	diagnosis, efficacy
P-Selectin	P-Selectin (SELP)	efficacy, safety
RET	Proto-oncogene tyrosine-protein kinase receptor Ret (RET)	efficacy, response to therapy
SAA	Serum amyloid A-1 protein (SAA1)	diagnosis
sE-Selectin	E-Selectin (SELE)	diagnosis, disease progression, efficacy
sICAM-1	Intercellular adhesion molecule 1 (ICAM1)	diagnosis, efficacy, prognosis
Siglec-3	Myeloid cell surface antigen CD33 (CD33)	efficacy
sLeptin R	Leptin receptor (LEPR)	prognosis
sL-Selectin	L-Selectin (SELL)	efficacy
SPARCL1	SPARC-like protein 1 (SPARCL1)	disease progression
SPINT2	Kunitz-type protease inhibitor 2 (SPINT2)	diagnosis
sTie-2	Angiopoietin-1 receptor, soluble (TEK)	efficacy
TECK	C-C motif chemokine 25 (CCL25)	disease progression
TIMP-3	Metalloproteinase inhibitor 3 (TIMP3)	diagnosis, prognosis
VEGF sR2	Vascular endothelial growth factor receptor 2 (KDR)	disease progression, efficacy, prognosis, response to therapy, safety
VEGF sR3	Vascular endothelial growth factor receptor 3 (FLT4)	diagnosis, disease progression, efficacy, prognosis
vWF	von Willebrand factor (VWF)	diagnosis, efficacy, prognosis

Supplementary Table 7: Proteins with a replicated pQTL that are targeted by a drug.
Annotation as drug target derived from Ingenuity Pathway Analysis (IPA) database.

Target	TargetFullName (EntrezGeneSymbol)	Targeted by drug(s)
AMPM2	Methionine aminopeptidase 2 (METAP2)	nitroxoline, beloranib, PPI-2458, …
Angiostatin	Angiostatin (PLG)	tenecteplase, PLAT, tranexamic acid, …
annexin I	Annexin A1 (ANXA1)	hydrocortisone, hydrocortisone/prednisone, hydrocortisone/mitoxantrone, …
Apo B	Apolipoprotein B (APOB)	mipomersen
bFGF	Fibroblast growth factor 2 (FGF2)	pentosan polysulfate, suradista, sucralfate, …
C1r	Complement C1r subcomponent (C1R)	SERPING1
C1s	Complement C1s subcomponent (C1S)	SERPING1
Carbonic anhydrase 6	Carbonic anhydrase 6 (CA6)	methazolamide, hydrochlorothiazide, acetazolamide, …
Carbonic anhydrase XIII	Carbonic anhydrase 13 (CA13)	methazolamide, hydrochlorothiazide, acetazolamide, …
Caspase-3	Caspase-3 (CASP3)	emricasan
Catalase	Catalase (CAT)	fomepizole
Coagulation Factor V	Coagulation Factor V (F5)	drotrecogin alfa, antithrombin alfa
Coagulation Factor VII	Coagulation Factor VII (F7)	nematode anticoagulant protein c2
ER	Estrogen receptor (ESR1)	17-alpha-ethinylestradiol, fulvestrant, beta-estradiol, …
FCG3B	Low affinity immunoglobulin gamma Fc region receptor III-B (FCGR3B)	IgG
Fibronectin	Fibronectin (FN1)	ocriplasmin
G-CSF	Granulocyte colony-stimulating factor (CSF3)	filgrastim
Heparin cofactor II	Heparin cofactor 2 (SERPIND1)	ardeparin, glucuronyl glucosamine glycan sulfate
IL-1b	Interleukin-1 beta (IL1B)	canakinumab, gevokizumab, canakinumab/INS, …
IL-6 sRa	Interleukin-6 receptor subunit alpha (IL6R)	tocilizumab
IL-7 Ra	Interleukin-7 receptor subunit alpha (IL7R)	recombinant human interleukin-7
IMDH1	Inosine-5'-monophosphate dehydrogenase 1 (IMPDH1)	thioguanine, VX-944, pegintron/ribavirin, …
IMDH2	Inosine-5'-monophosphate dehydrogenase 2 (IMPDH2)	thioguanine, VX-944, pegintron/ribavirin, …
IP-10	C-X-C motif chemokine 10 (CXCL10)	MDX-1100
IR	Insulin receptor (INSR)	insulin detemir, INS, canakinumab/INS, …
Met	Hepatocyte growth factor receptor (MET)	crizotinib, tivantinib, cabozantinib, …

MMP-1	Interstitial collagenase (MMP1)	marimastat
MMP-12	Macrophage metalloelastase (MMP12)	marimastat
MMP-7	Matrilysin (MMP7)	marimastat
MMP-8	Neutrophil collagenase (MMP8)	marimastat
MP2K2	Dual specificity mitogen-activated protein kinase kinase 2 (MAP2K2)	selumetinib, trametinib, dabrafenib/trametinib, …
NPS-PLA2	Phospholipase A2, membrane associated (PLA2G2A)	varespladib methyl, varespladib, indomethacin, …
PDE5A	cGMP-specific 3',5'-cyclic phosphodiesterase (PDE5A)	dyphylline, nitroglycerin, udenafil, …
PDGF Rb	Platelet-derived growth factor receptor beta (PDGFRB)	nilotinib, dasatinib, sunitinib, …
PDK1	[Pyruvate dehydrogenase (acetyl-transferring)] kinase isozyme 1, mitochondrial (PDK1)	dichloroacetic acid
Plasminogen	Plasminogen (PLG)	tenecteplase, PLAT, tranexamic acid, …
Prekallikrein	Plasma kallikrein (KLKB1)	aprotinin, ecallantide
RET	Proto-oncogene tyrosine-protein kinase receptor Ret (RET)	sunitinib, motesanib, cabozantinib, …
Siglec-3	Myeloid cell surface antigen CD33 (CD33)	arsenic trioxide/gemtuzumab ozogamicin/tretinoin, gemtuzumab ozogamicin
sLeptin R	Leptin receptor (LEPR)	recombinant-methionyl human leptin
sTie-2	Angiopoietin-1 receptor, soluble (TEK)	cabozantinib, regorafenib, ponatinib, …
VEGF sR2	Vascular endothelial growth factor receptor 2 (KDR)	AEE 788, sunitinib, cediranib, …
VEGF sR3	Vascular endothelial growth factor receptor 3 (FLT4)	sunitinib, pazopanib, axitinib, …

Supplementary Table 8: Metabolomics QTLs

List of 14 mQTLs from metabolomics GWAS that overlap with pQTLs reported here; only the lead target protein (SomaLogic annotation) for each locus is listed, together with the locus number; mQTLs are limited to the strongest association signal and requiring $p < 10^{-8}$ and p-gain > 10^4; all associations for each locus, and also further less significant associations can be obtained from http://gwas.eu.

Target	Locus	SNP	Chr	Position	P-value	P-gain	Metabolite/-ratio
sE-Selectin	28	rs651007	9	136,153,875	7.0E-34	9.0E+13	ADSGEGDFXAEGGGVR / ADpSGEGDFXAEGGGVR[1]
DC-SIGN	36	rs505922	9	136,149,229	4.5E-34	1.7E+21	ADSGEGDFXAEGGGVR / ADpSGEGDFXAEGGGVR
Coagulation Factor XI	96	rs5030062	3	186,454,180	5.9E-13	-	bradykinin, des-arg(9)
Factor H	111	rs10737680	1	196,679,455	2.9E-09	-	HWESASXX
Mn SOD	113	rs2545801	5	176,841,339	9.6E-18	-	bradykinin, des-arg(9)
SAA	121	rs4150579	11	18,357,180	2.6E-26	1.4E+05	3-(4-hydroxyphenyl)lactate / alpha-hydroxyisovalerate
IGF-II receptor	127	rs2297363	6	160,506,462	5.2E-09	-	isobutyrylcarnitine
Prekallikrein	138	rs1511802	4	187,150,806	4.3E-28	-	bradykinin, des-arg(9)
PPID	156	rs8396	4	159,630,817	3.3E-59	4.2E+27	octanoylcarnitine / X-13435
Kininogen, HMW	288	rs3856930	3	186,458,322	4.2E-11	-	bradykinin, des-arg(9)
IGF-II receptor	315	rs9295123	6	160,570,114	1.7E-09	-	isobutyrylcarnitine
sE-Selectin	354	rs8176720	9	136,132,873	2.5E-11	2.3E+04	ADpSGEGDFXAEGGGVR / leucylalanine
IGF-II receptor	358	rs12202350	6	160,379,096	5.1E-18	-	isobutyrylcarnitine
AK1A1	441	rs6662572	1	46,146,812	6.5E-10	-	erythritol

[1]ADSGEGDFXAEGGGVR / ADpSGEGDFXAEGGGVR is a measure for fibrinogen A-alpha phosphorylation

Supplementary Table 9: Overlapping hits with disease GWAS associations.

List of overlapping disease GWAS hits (GWAS catalogue via SNiPA) and publicly available summary statistics data from 14 large disease GWAS consortia (p-value cut-off $<10^{-9}$); curated to eliminate duplicated entries and non-disease endpoints, such as height, response to medication, and lipid traits; only the lead target protein (SomaLogic annotation) for each locus is listed, together with the locus number, further details can be obtained online.

Target	Locus	SNP	Chr	Position	Curated Trait	Source
IL-6 sRa	2	rs4129267	1	154,426,264	Asthma	GWAS catalogue
IL-6 sRa	2	rs4129267	1	154,426,264	Pulmonary function	GWAS catalogue
MSP	4	rs9858542	3	49,701,983	Crohn's disease	GWAS catalogue
MSP	4	rs9858542	3	49,701,983	Educational attainment	GWAS catalogue
MSP	4	rs9858542	3	49,701,983	Inflammatory bowel disease	GWAS catalogue
MSP	4	rs9858542	3	49,701,983	Primary sclerosing cholangitis	GWAS catalogue
MSP	4	rs9858542	3	49,701,983	Ulcerative colitis	GWAS catalogue
sLeptin R	5	rs17415296	1	66,099,013	Fasting plasma glucose (childhood)	GWAS catalogue
Siglec-3	7	rs12459419	19	51,728,477	Alzheimer's disease	GWAS catalogue
ARTS1	11	rs26496	5	96,137,882	Ankylosing spondylitis	GWAS catalogue
MICA	13	rs2844511	6	31,389,784	Rheumatoid Arthritis	GWAS consortia
ARTS1	14	rs17482078	5	96,118,866	Behcet's disease	GWAS catalogue
FCG2A/B	16	rs7551957	1	161,470,042	Inflammatory bowel disease	GWAS catalogue
FCG2A/B	16	rs7551957	1	161,470,042	Kawasaki disease	GWAS catalogue
FCG2A/B	16	rs7551957	1	161,470,042	Systemic lupus erythematosus	GWAS catalogue
FCG2A/B	16	rs7551957	1	161,470,042	Ulcerative colitis	GWAS catalogue
IL-18 Ra	19	rs2058622	2	102,985,424	Atopic dermatitis	GWAS catalogue
IL-18 Ra	19	rs2058622	2	102,985,424	Celiac disease	GWAS catalogue
IL-18 Ra	19	rs2058622	2	102,985,424	Crohn's disease	GWAS catalogue
IL-18 Ra	19	rs2058622	2	102,985,424	Inflammatory bowel disease	GWAS catalogue
sE-Selectin	28	rs651007	9	136,153,875	Heart disease	GWAS catalogue
sE-Selectin	28	rs651007	9	136,153,875	Stroke	GWAS catalogue
sE-Selectin	28	rs651007	9	136,153,875	Venous thromboembolism	GWAS catalogue
GPC5	37	rs342706	13	92,417,058	Lung cancer	GWAS catalogue
IL-1 R4	44	rs12712135	2	102,930,948	Leprosy	GWAS catalogue
ILT-4	48	rs103294	19	54,797,848	Prostate cancer	GWAS catalogue
CPNE1	50	rs2425143	20	34,350,666	Obesity related traits	GWAS consortia
G-CSF	54	rs5167	19	45,448,465	Alzheimer's disease	GWAS consortia

FCRL3	64	rs7522061	1	157,668,390	Graves' disease	GWAS catalogue
FCRL3	64	rs7522061	1	157,668,390	Multiple sclerosis	GWAS catalogue
Cadherin-5	69	rs8176749	9	136,131,188	Intraocular pressure	GWAS catalogue
GRN	71	rs646776	1	109,818,530	Heart disease	GWAS catalogue
GRN	71	rs646776	1	109,818,530	Stroke	GWAS catalogue
TXD12	95	rs11715835	3	48,770,732	Inflammatory bowel disease	GWAS consortia
MICB	105	rs3094005	6	31,465,047	Schizophrenia	GWAS consortia
Protein C	106	rs867186	20	33,764,554	Amyotrophic lateral sclerosis	GWAS catalogue
MMP-12	107	rs17368582	11	102,738,075	Local histogram emphysema pattern	GWAS catalogue
Factor H	111	rs10737680	1	196,679,455	Age-related macular degeneration	GWAS catalogue
SAA	121	rs4150579	11	18,357,180	Pancreatic cancer	GWAS catalogue
MSP	130	rs13063312	3	48,661,985	Inflammatory bowel disease	GWAS consortia
MSP	130	rs13063312	3	48,661,985	Ulcerative colitis	GWAS consortia
C4b	137	rs2280774	6	31,928,691	Rheumatoid Arthritis	GWAS consortia
Factor B	142	rs4151669	6	31,915,144	Rheumatoid Arthritis	GWAS consortia
Cathepsin B	145	rs3947	8	11,702,375	Parkinson's disease	GWAS catalogue
C34 gp41 HIV Fragment	159	rs387608	6	31,941,557	Rheumatoid Arthritis	GWAS consortia
Properdin	177	rs7529589	1	196,658,279	Age-related macular degeneration	GWAS catalogue
MICB	180	rs3132645	6	30,409,249	Schizophrenia	GWAS consortia
MICB	198	rs2248372	6	31,446,466	Rheumatoid Arthritis	GWAS consortia
MICB	199	rs3129682	6	29,283,672	Barrett's esophagus	GWAS catalogue
MICB	199	rs3129682	6	29,283,672	Schizophrenia	GWAS consortia
Factor B	200	rs550513	6	31,920,687	Rheumatoid Arthritis	GWAS consortia
Cathepsin S	201	rs1336900	1	150,679,033	Fat body mass	GWAS catalogue
MICB	206	rs1794282	6	32,666,526	Rheumatoid Arthritis	GWAS consortia
MICB	206	rs1794282	6	32,666,526	Schizophrenia	GWAS consortia
SAA	233	rs16935432	11	18,470,487	Amyotrophic lateral sclerosis	GWAS catalogue
C4	235	rs8283	6	32,083,300	Rheumatoid Arthritis	GWAS consortia
CPNE1	244	rs1204656	20	33,982,846	Obesity related traits	GWAS consortia
MICA	245	rs3134942	6	32,168,771	Rheumatoid Arthritis	GWAS consortia
IL-23 R	251	rs11209026	1	67,705,958	Ankylosing spondylitis	GWAS catalogue
IL-23 R	251	rs11209026	1	67,705,958	Crohn's disease	GWAS catalogue
IL-23 R	251	rs11209026	1	67,705,958	Inflammatory bowel disease	GWAS catalogue
IL-23 R	251	rs11209026	1	67,705,958	Psoriasis	GWAS catalogue
IL-23 R	251	rs11209026	1	67,705,958	Ulcerative colitis	GWAS catalogue
MICB	253	rs33932084	6	28,268,824	Schizophrenia	GWAS consortia

MICB	260	rs2534674	6	31,465,103	Rheumatoid Arthritis	GWAS consortia
MICA	271	rs4418214	6	31,391,401	HIV-1 susceptibility, HIV-1 control	GWAS catalogue
Cystatin C	293	rs6036478	20	23,611,359	Chronic kidney disease	GWAS catalogue
RET	294	rs2795502	10	43,340,662	Progressive supranuclear palsy	GWAS catalogue
RUXF	306	rs4420638	19	45,422,946	Age-related macular degeneration	GWAS catalogue
RUXF	306	rs4420638	19	45,422,946	Alzheimer's disease	GWAS catalogue
RUXF	306	rs4420638	19	45,422,946	Longevity	GWAS catalogue
C7	314	rs7713972	5	40,552,474	Crohn's disease	GWAS consortia
a1-Antitrypsin	320	rs4905179	14	94,795,492	Breast size	GWAS catalogue
MICB	330	rs35555795	6	26,509,382	Schizophrenia	GWAS consortia
NKG2D	332	rs2255336	12	10,532,326	Obesity-related traits	GWAS catalogue
MICA	342	rs2523987	6	30,079,993	Rheumatoid Arthritis	GWAS consortia
MICA	342	rs2523987	6	30,079,993	Schizophrenia	GWAS consortia
C4	372	rs2239805	6	32,411,376	Rheumatoid Arthritis	GWAS consortia
C4	372	rs2239805	6	32,411,376	Schizophrenia	GWAS consortia
TARC	377	rs983545	3	16,959,652	Primary biliary cirrhosis	GWAS catalogue
C4b	379	rs204888	6	32,089,142	Rheumatoid Arthritis	GWAS consortia
granzyme A	387	rs2853928	6	31,257,511	Schizophrenia	GWAS consortia
annexin I	436	rs4745216	9	75,744,157	Schizophrenia	GWAS catalogue

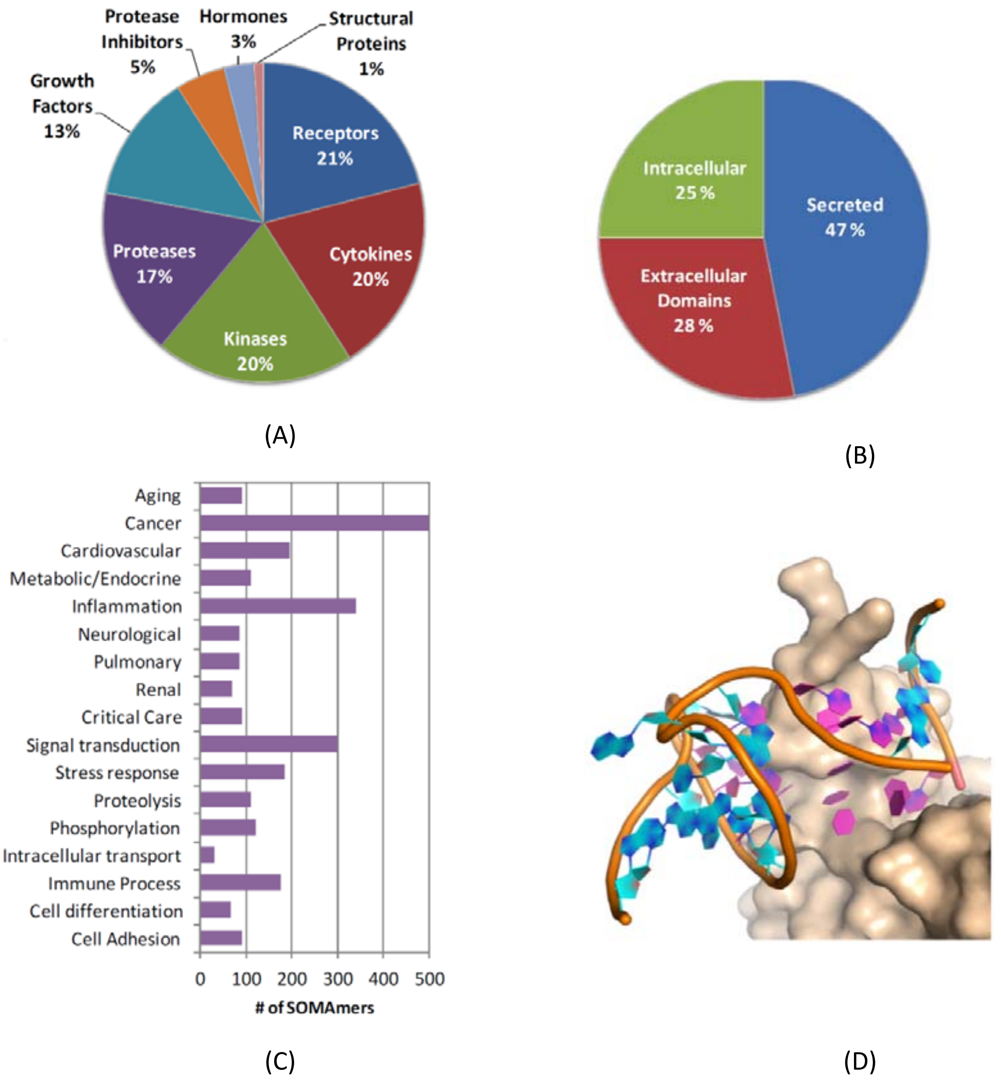

Supplementary Figure 1: Protein types and classes covered by the SOMAscan panel.

The SOMAscan panel used in this study comprises 1,129 aptamers (**Supplementary** Data 2). These bind proteins in numerous disease areas and physiological processes (A); The aptamers have been generated to target various classes of proteins (B), covering secreted, intracellular and extracellular domains of proteins (C); Example of an X-ray crystal structure of an aptamer bound to a protein (D); Technical details of the SOMAscan assay are provided in the SomaLogic Technical Whitepaper (available at http://www.somalogic.com/Resources/White-Papers-and-Tech-Notes.aspx).

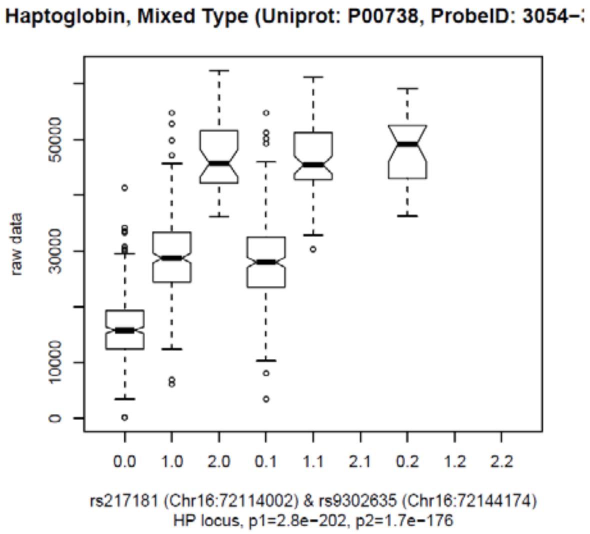
Haptoglobin, Mixed Type (Uniprot: P00738, ProbeID: 3054-
raw data
0
10000
30000
50000
0.0 1.0 2.0 0.1 1.1 2.1 0.2 1.2 2.2
rs217181 (Chr16:72114002) & rs9302635 (Chr16:72144174)
HP locus, p1=2.8e−202, p2=1.7e−176

(A)

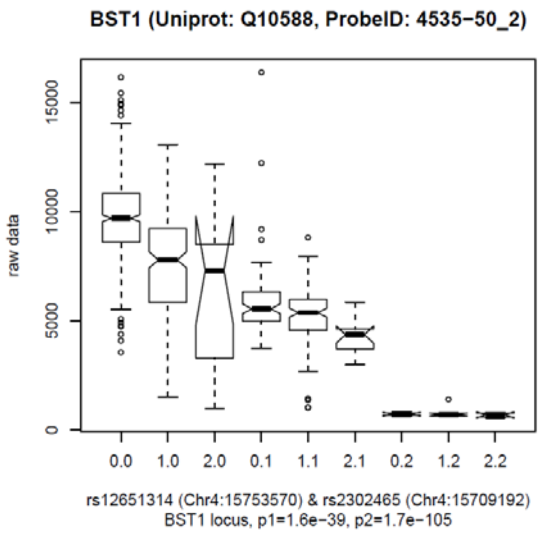
BST1 (Uniprot: Q10588, ProbeID: 4535−50_2)
raw data
0
5000
10000
15000
0.0 1.0 2.0 0.1 1.1 2.1 0.2 1.2 2.2
rs12651314 (Chr4:15753570) & rs2302465 (Chr4:15709192)
BST1 locus, p1=1.6e−39, p2=1.7e−105

(B)

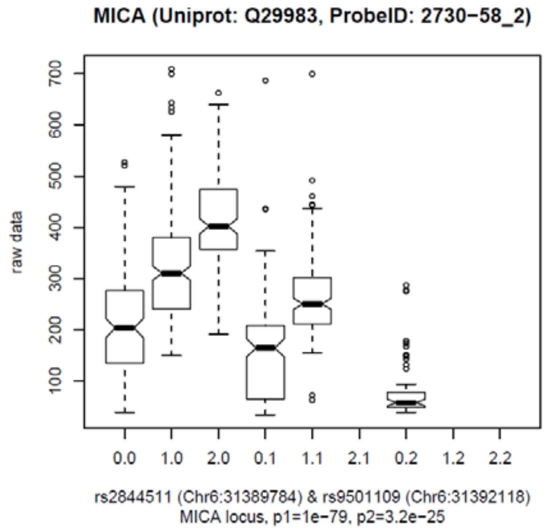
MICA (Uniprot: Q29983, ProbeID: 2730−58_2)
raw data
100
200
300
400
500
600
700
0.0 1.0 2.0 0.1 1.1 2.1 0.2 1.2 2.2
rs2844511 (Chr6:31389784) & rs9501109 (Chr6:31392118)
MICA locus, p1=1e−79, p2=3.2e−25

(C)

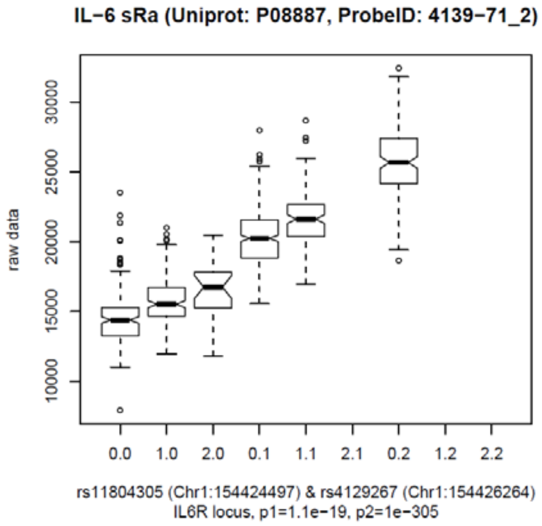
IL−6 sRa (Uniprot: P08887, ProbeID: 4139−71_2)
raw data
10000
15000
20000
25000
30000
0.0 1.0 2.0 0.1 1.1 2.1 0.2 1.2 2.2
rs11804305 (Chr1:154424497) & rs4129267 (Chr1:154426264)
IL6R locus, p1=1.1e−19, p2=1e−305

(D)

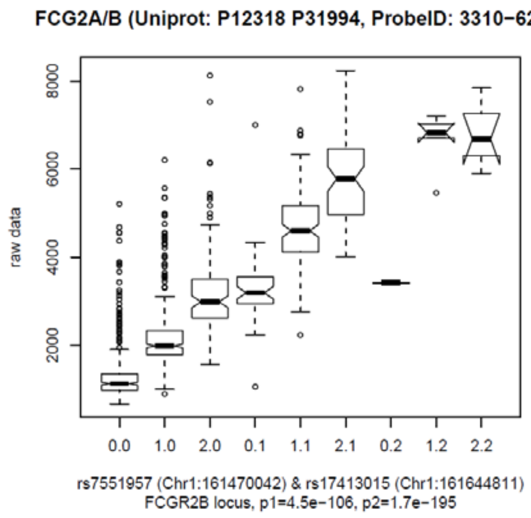

(E)

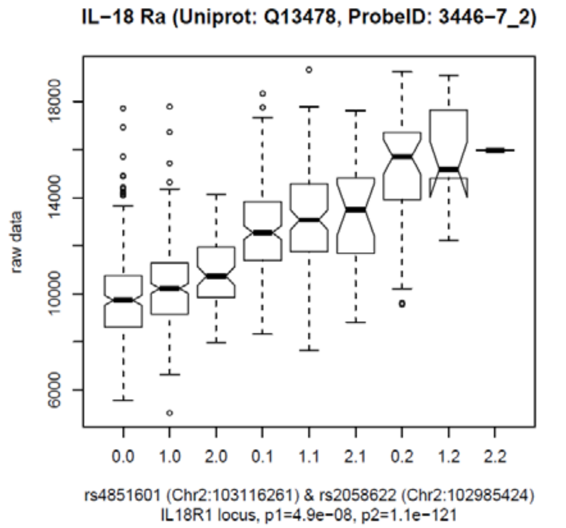

(F)

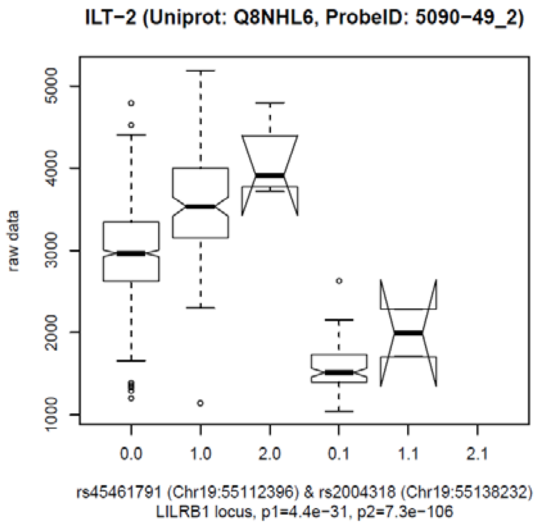

(G)

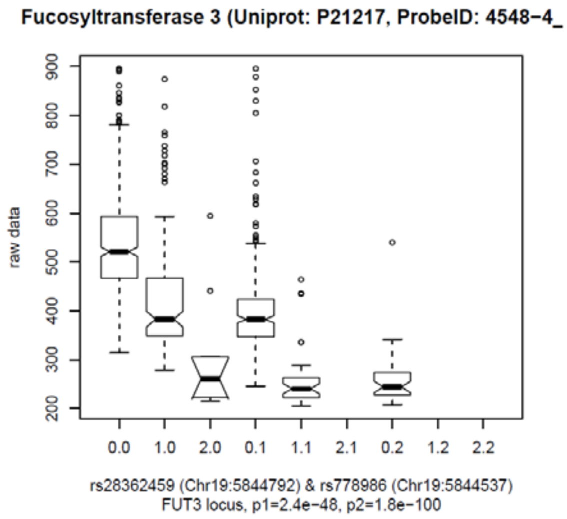

(H)

Supplementary Figure 2: Examples of proteins associated with multiple genetic variants.
Boxplots of protein levels (unscaled data) as a function of two genotypes, labelled by the number of the respective minor allele variants; P-values are for the contribution of each variant in a combined linear model with all covariates and unscaled protein levels as dependent variable; Outliers that were more than four standard deviations from the mean were removed here to optimize the plot area.

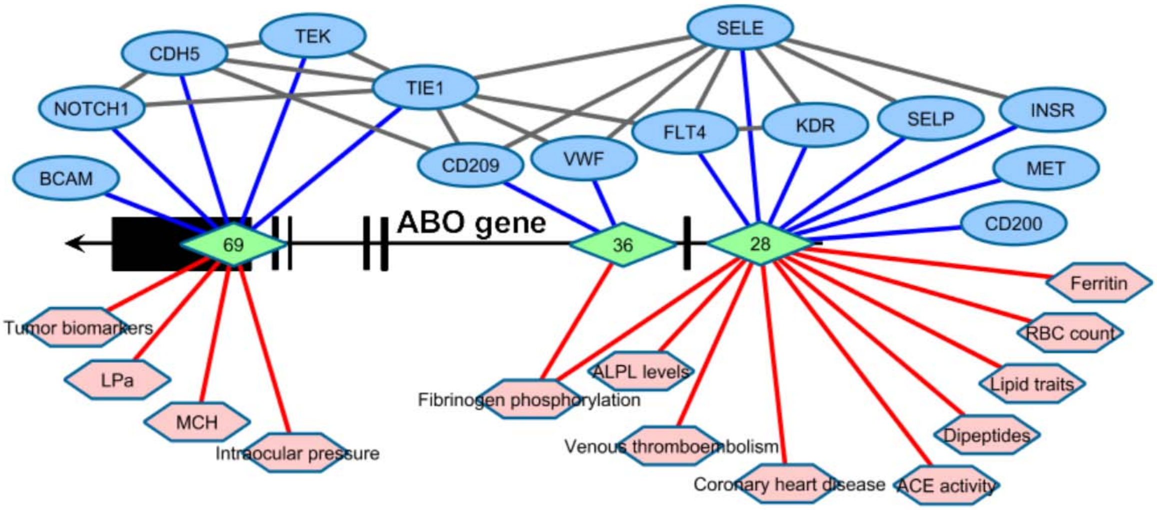

Supplementary Figure 3: Network view of the ABO locus.

Replicated associations of the three strongest variants at the ABO locus (gene structure in black) with protein plasma levels (green diamonds & blue lines); Partial correlations between proteins (blue ellipses & grey lines); previously reported GWAS associations (pink hexagons & red lines).

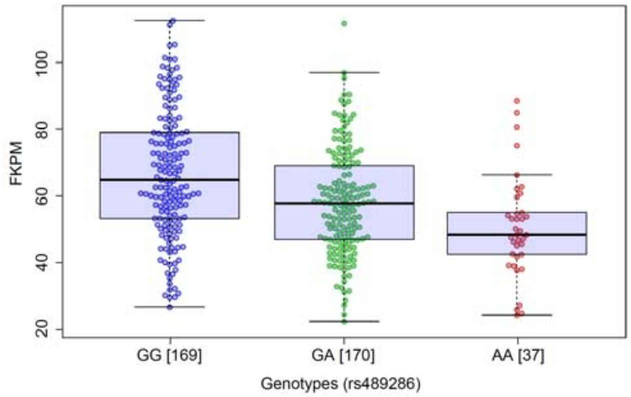

(A)

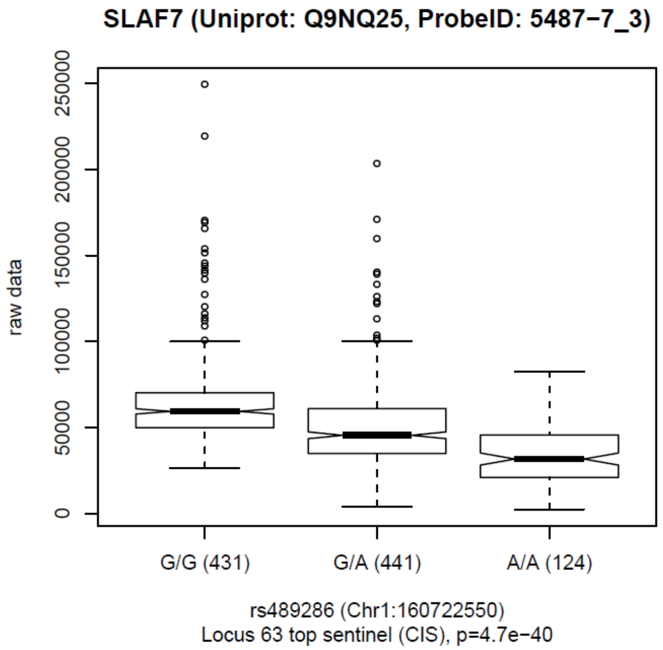

(B)

Supplementary Figure 4: SLAMF7 protein and gene expression.
Boxplots of SLAMF7 mRNA expression levels in lymphoblastoid cells (A) and protein levels of SLAMF7 (aka SLAF7) in blood (B).

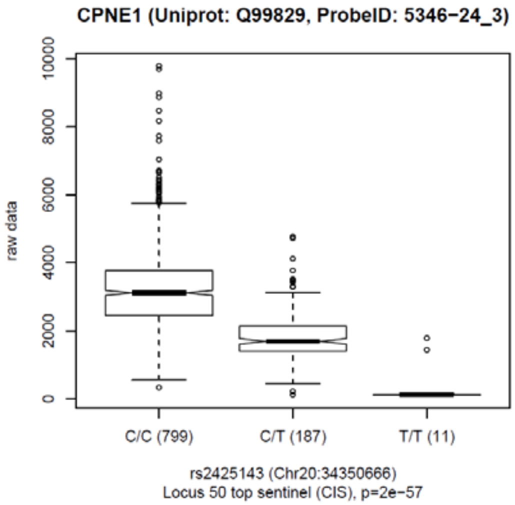

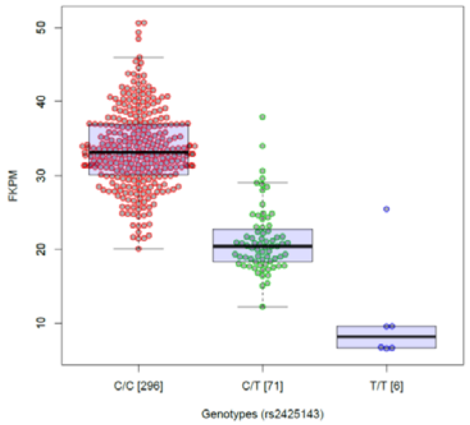

(A) (B)

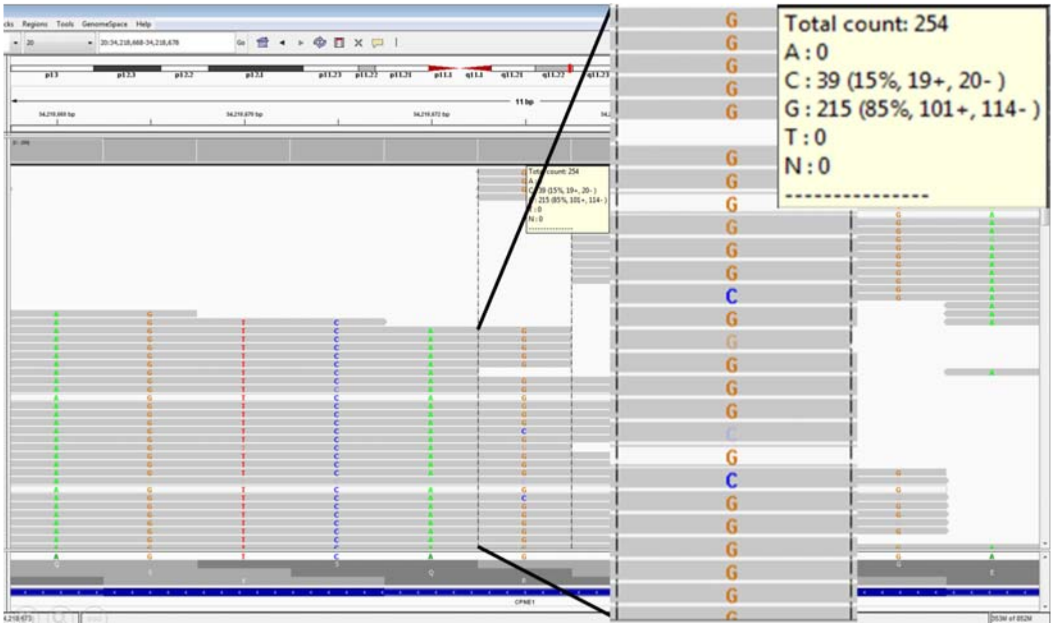

Supplementary Figure 5: Allele specific mRNA expression of CPNE1.

Near total protein ablation of CPNE1 protein levels in rs2425143_T homozygotes (A), confirmed by mRNA expression (normalized read counts, FPKM) in lymphoblastoid cell lines of the 1000 Genomes Project as a function of rs2425143 genotype (B), and an example of allele specific mRNA expression analysis (CPNE1 read-alignments and variant calls for a rs2425143 heterozygote; the highlighted variant is located inside the CPNE1 transcript) (C).

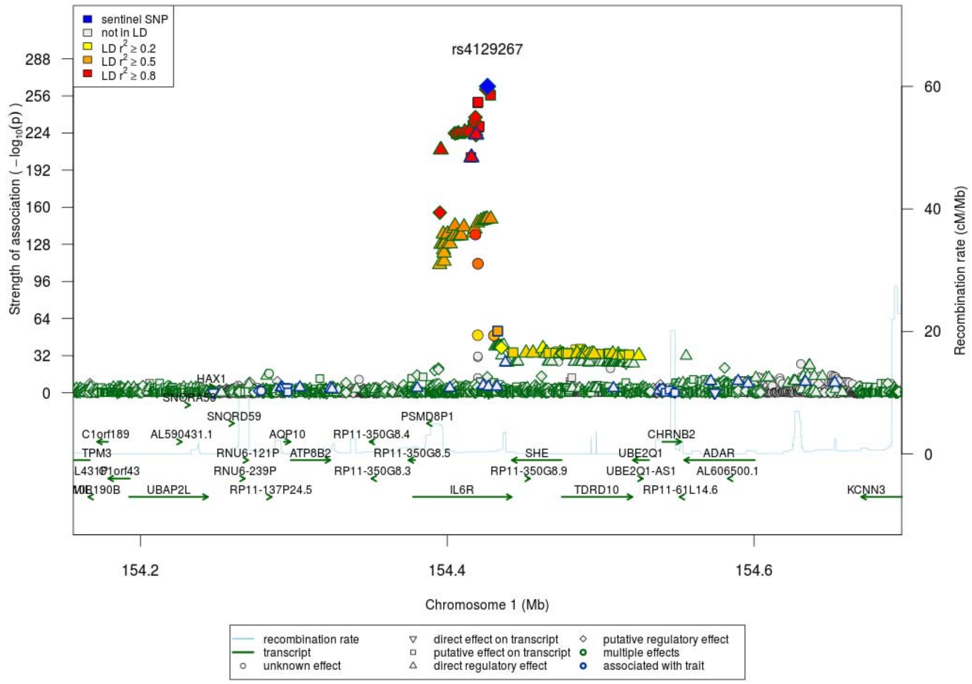

Supplementary Figure 6: Regional association plots for all significant *cis*- and *trans*-associations. Regional association plots using 1000-Genomes imputed genotypes in KORA for all 539 SNP-probe associations are provided as PNG files. The corresponding raw association data is provided as ASCII files. Plots and data can be accessed online.

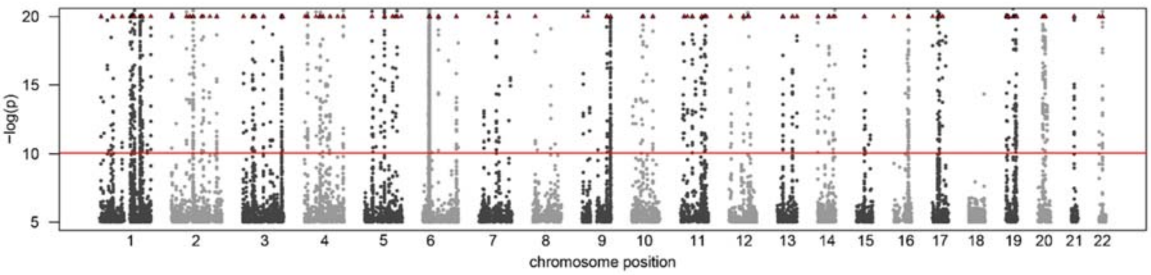

Supplementary Figure 7: Manhattan plot.

Red line: Bonferroni level of significance, associations with $p<10^{-20}$ are marked by a red triangle.

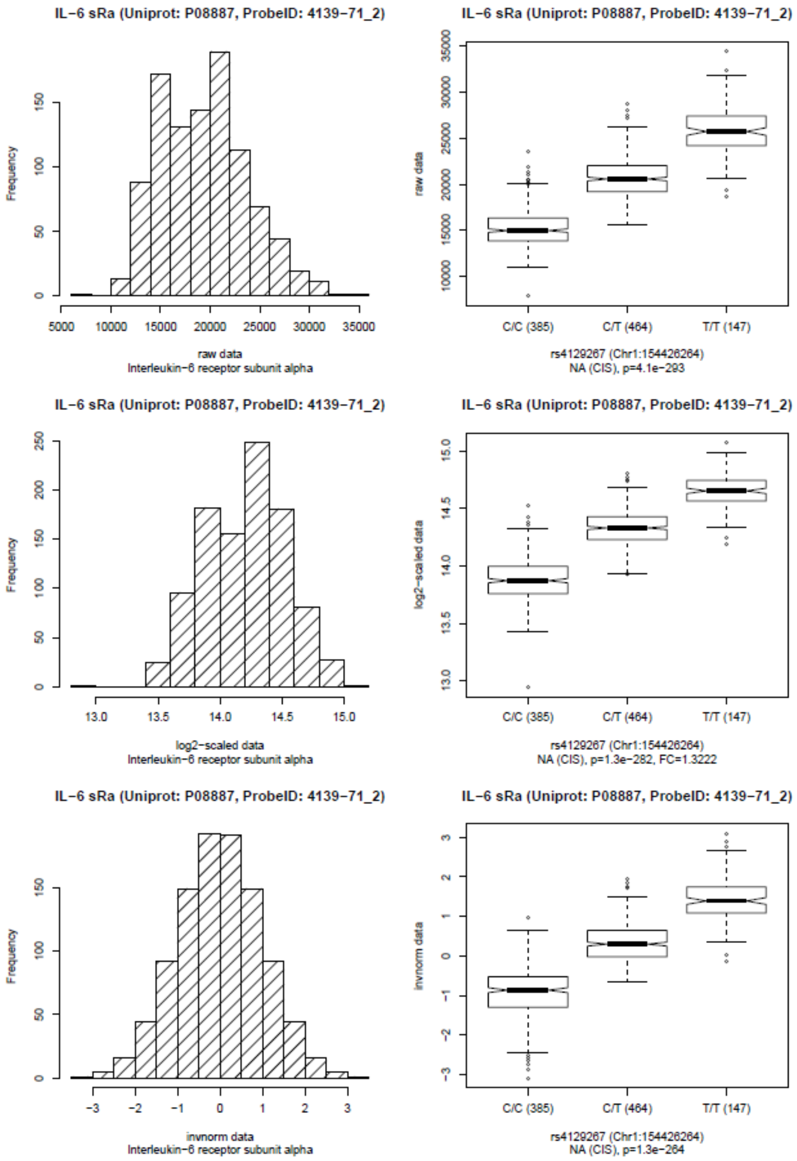

Supplementary Figure 8: Histograms and boxplots for all significant *cis*- and *trans*-associations.

Histograms and boxplots using three different scaling methods (raw, log2, inverse-normal) with association data and allele counts are provided as a separate PDF file for all significant 539 SNP-protein associations in KORA and all 462 replication attempts in QMDiab. These plots can also be accessed online.

SNiPAcard

Block annotations

Block info

genomic range	chr1:47,257,669-47,424,594
block size	166,926 bp
variant count	100 variants

Basic features

Conservation/deleteriousness		Linked genes	
phyloP	μ = -0.332 [-3.743 – 0.859]	gene(s) hit or close-by	CYP4A11, CYP4B1, CYP4Z2P
phastCons	μ = 0.101 [0 – 0.808]	eQTL gene(s)	CYP4B1, HPDL
GERP++	μ = -0.225 [-6.04 – 3.35]	potentially regulated gene(s)	ATPAF1, CYP4B1, CYP4Z1, MKNK1
CADD score	μ = 3.807 [0.04 – 23.9]	disease gene(s)	ATPAF1

Trait annotations

Variant association

trait	min(p-value)	source DB	source entry/link	variant(s)
Blood metabolite levels	$<2.00\times10^{-61}$	GWAS Catalog	24816252	2
Blood metabolite ratios	$<8.00\times10^{-86}$	GWAS Catalog	24816252	1
Metabolic traits	$<5.00\times10^{-32}$	GWAS Catalog	21886157	1

Supplementary Figure 9: Example screenshot of a SNiPA annotation

SNiPA annotations for 435 out of 451 loci (sentinel SNPs and proxy variants in linkage disequilibrium $r^2 > 0.8$; 16 sentinel SNPs were not available in the SNiPA database); SNiPA annotations for all 539 SNP-probe associations are provided as PDF files and can be accessed online. Continually updated annotations can also be retrieved using the SNiPA web server at http://snipa.org by entering the sentinel SNP identifier into the block-annotation tool.

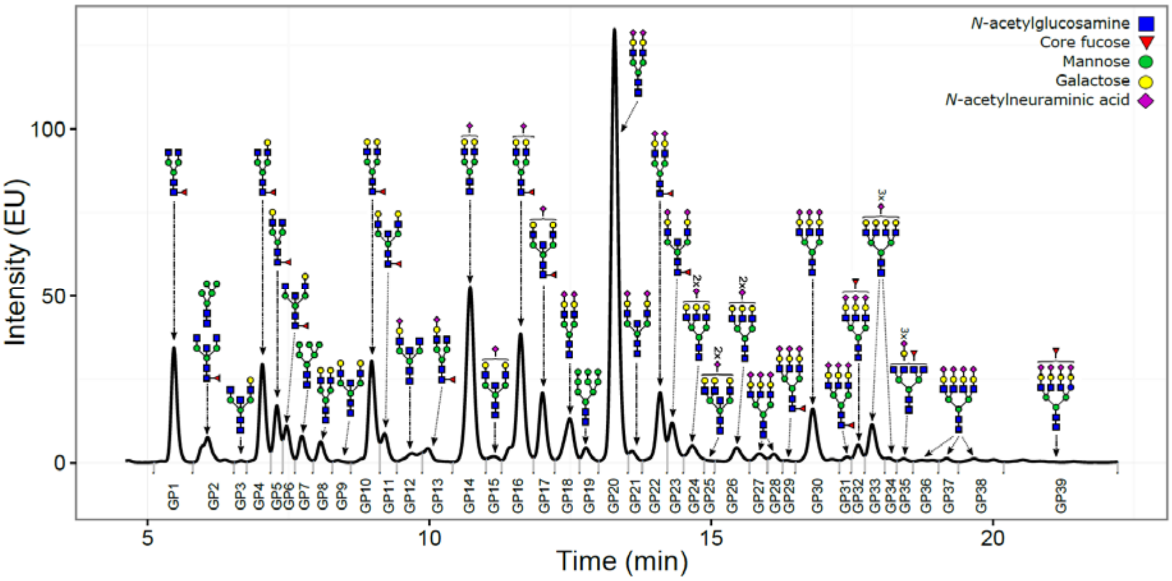

Supplementary Figure 10: Representative chromatogram of a total plasma N-glycome.

Fluorescently labelled plasma N-glycans were separated by HILIC-UPLC into 39 peaks (GP1-GP39). The glycan content in each peak was assigned as determined previously [22]. The amount of glycan species in each peak was expressed as % of total integrated area.

SUPPLEMENTARY REFERENCES

1. Melzer, D. *et al.* A genome-wide association study identifies protein quantitative trait loci (pQTLs). *PLoS Genet.* **4,** e1000072 (2008).
2. Lourdusamy, A. *et al.* Identification of cis-regulatory variation influencing protein abundance levels in human plasma. *Hum. Mol. Genet.* **21,** 3719–3726 (2012).
3. Johansson, Å. *et al.* Identification of genetic variants influencing the human plasma proteome. *Proc. Natl. Acad. Sci. U. S. A.* **110,** 4673–8 (2013).
4. Kim, S. *et al.* Influence of Genetic Variation on Plasma Protein Levels in Older Adults Using a Multi-Analyte Panel. *PLoS One* **8,** (2013).
5. Liu, Y. *et al.* Quantitative variability of 342 plasma proteins in a human twin population. *Mol. Syst. Biol.* **11,** 786 (2015).
6. Enroth, S., Johansson, A., Enroth, S. B. & Gyllensten, U. Strong effects of genetic and lifestyle factors on biomarker variation and use of personalized cutoffs. *Nat. Commun.* **5,** 4684 (2014).
7. Carrasquillo, M. M. *et al.* Genome-wide screen identifies rs646776 near sortilin as a regulator of progranulin levels in human plasma. *Am. J. Hum. Genet.* **87,** 890–7 (2010).
8. van Dongen, J. *et al.* The contribution of the functional IL6R polymorphism rs2228145, eQTLs and other genome-wide SNPs to the heritability of plasma sIL-6R levels. *Behav. Genet.* **44,** 368–382 (2014).
9. Barbalic, M. *et al.* Large-scale genomic studies reveal central role of ABO in sP-selectin and sICAM-1 levels. *Hum. Mol. Genet.* **19,** 1863–72 (2010).
10. Ho, J. E. *et al.* Common genetic variation at the IL1RL1 locus regulates IL-33/ST2 signaling. *J. Clin. Invest.* **123,** 4208–18 (2013).
11. Heid, I. M. *et al.* Clear detection of ADIPOQ locus as the major gene for plasma adiponectin: Results of genome-wide association analyses including 4659 European individuals. *Atherosclerosis* **208,** 412–420 (2010).
12. Kaplan, R. C. *et al.* A genome-wide association study identifies novel loci associated with circulating IGF-I and IGFBP-3. *Hum. Mol. Genet.* **20,** 1241–51 (2011).
13. Zabaneh, D. *et al.* Genetic Variants Associated with von Willebrand Factor Levels in Healthy Men and Women Identified Using the HumanCVD BeadChip. *Ann. Hum. Genet.* **75,** 456–467 (2011).
14. Qi, L. *et al.* Genetic variants in ABO blood group region, plasma soluble E-selectin levels and risk of type 2 diabetes. *Hum. Mol. Genet.* **19,** 1856–1862 (2010).
15. Karakas, M. *et al.* A Variant In the Abo Gene Explains the Variation in Soluble E-Selectin Levels-Results from Dense Genotyping in Two Independent Populations. *PLoS One* **7,** 3–8 (2012).
16. Paré, G. *et al.* Genome-wide association analysis of soluble ICAM-1 concentration reveals novel associations at the NFKBIK, PNPLA3, RELA, and SH2B3 loci. *PLoS Genet.* **7,** e1001374 (2011).
17. Marzi, C. *et al.* Genome-wide association study identifies two novel regions at 11p15.5-p13 and 1p31 with major impact on acute-phase serum amyloid A. *PLoS Genet.* **6,** e1001213 (2010).
18. Yang, Q., Kathiresan, S., Lin, J.-P., Tofler, G. H. & O'Donnell, C. J. Genome-wide association and linkage analyses of hemostatic factors and hematological phenotypes in the Framingham Heart Study. *BMC Med. Genet.* **8 Suppl 1,** S12 (2007).
19. Froguel, P. *et al.* A genome-wide association study identifies rs2000999 as a strong genetic determinant of circulating haptoglobin levels. *PLoS One* **7,** e32327 (2012).
20. Hwang, S.-J., Yang, Q., Meigs, J. B., Pearce, E. N. & Fox, C. S. A genome-wide association for kidney function and endocrine-related traits in the NHLBI's Framingham Heart Study. *BMC*

Med. Genet. **8 Suppl 1,** S10 (2007).
21. Tang, W. *et al.* Genome-wide association study identifies novel loci for plasma levels of protein C: the ARIC study. *Blood* **116,** 5032–6 (2010).
22. Saldova, R. *et al.* Association of N-glycosylation with breast carcinoma and systemic features using high-resolution quantitative UPLC. *J. Proteome Res.* **13,** 2314–2327 (2014).
